# Working memory capacity and fluid abilities: the more difficult the item, the more more is better

**DOI:** 10.3389/fpsyg.2014.00239

**Published:** 2014-03-21

**Authors:** Daniel R. Little, Stephan Lewandowsky, Stewart Craig

**Affiliations:** ^1^Melbourne School of Psychological Sciences, The University of MelbourneMelbourne, VIC, Australia; ^2^School of Experimental Psychology, University of BristolBristol, UK; ^3^School of Psychology, The University of Western AustraliaCrawley, WA, Australia

**Keywords:** Raven's progressive matrices, working memory capacity

## Abstract

The relationship between fluid intelligence and working memory is of fundamental importance to understanding how capacity-limited structures such as working memory interact with inference abilities to determine intelligent behavior. Recent evidence has suggested that the relationship between a fluid abilities test, Raven's Progressive Matrices, and working memory capacity (WMC) may be invariant across difficulty levels of the Raven's items. We show that this invariance can only be observed if the overall correlation between Raven's and WMC is low. Simulations of Raven's performance revealed that as the overall correlation between Raven's and WMC increases, the item-wise point bi-serial correlations involving WMC are no longer constant but increase considerably with item difficulty. The simulation results were confirmed by two studies that used a composite measure of WMC, which yielded a higher correlation between WMC and Raven's than reported in previous studies. As expected, with the higher overall correlation, there was a significant positive relationship between Raven's item difficulty and the extent of the item-wise correlation with WMC.

## Introduction

There is no doubt that working memory (WM), the architecture responsible for manipulation and retention of information over brief periods of time, is a core component of human cognition. In particular, people's working-memory capacity (WMC) shares around 50% of the variance with general fluid intelligence (Kane et al., 2005) and is also predictive of performance in numerous reasoning tasks and other measures of higher cognitive ability. However, there is some dispute about the exact nature of the relationship between WMC and one important assay of fluid intelligence, Raven's Progressive Matrices (e.g., Raven et al., 2000). Raven's test has arguably gathered more attention in the cognitive literature than any other psychometric assay of fluid intelligence, largely because it is an induction task *par excellence* that can be modeled computationally (see e.g., Carpenter et al., 1990; Verguts et al., 2000; Rasmussen and Eliasmith, 2011). The relationship between fluid intelligence and working memory is of fundamental importance to understanding how capacity-limited structures such as working memory interact with inference abilities to determine intelligent behavior.

Raven's test is designed such that items differ considerably in difficulty, with easy items—presented early in the test—solvable by more than 90% of participants and the hardest items—presented last—being solvable by fewer than 10% of participants. In light of the typically strong correlation between measures of WMC and overall performance on Raven's, intuition might dictate that this correlation should be greatest for the more difficult items but nearly absent for the easy items—after all, if 90% of all people succeed on the easy problems, then surely even a modest WM capacity should suffice for those items, resulting in a low or nil correlation with WMC. It is only as items become more difficult that greater WMC is required for their solution, thus contributing to a higher correlation between performance on those items and WMC. Indeed, Carpenter et al. (1990) presented a computational model of Raven's performance that embodied this intuition.

**Figure 1:**
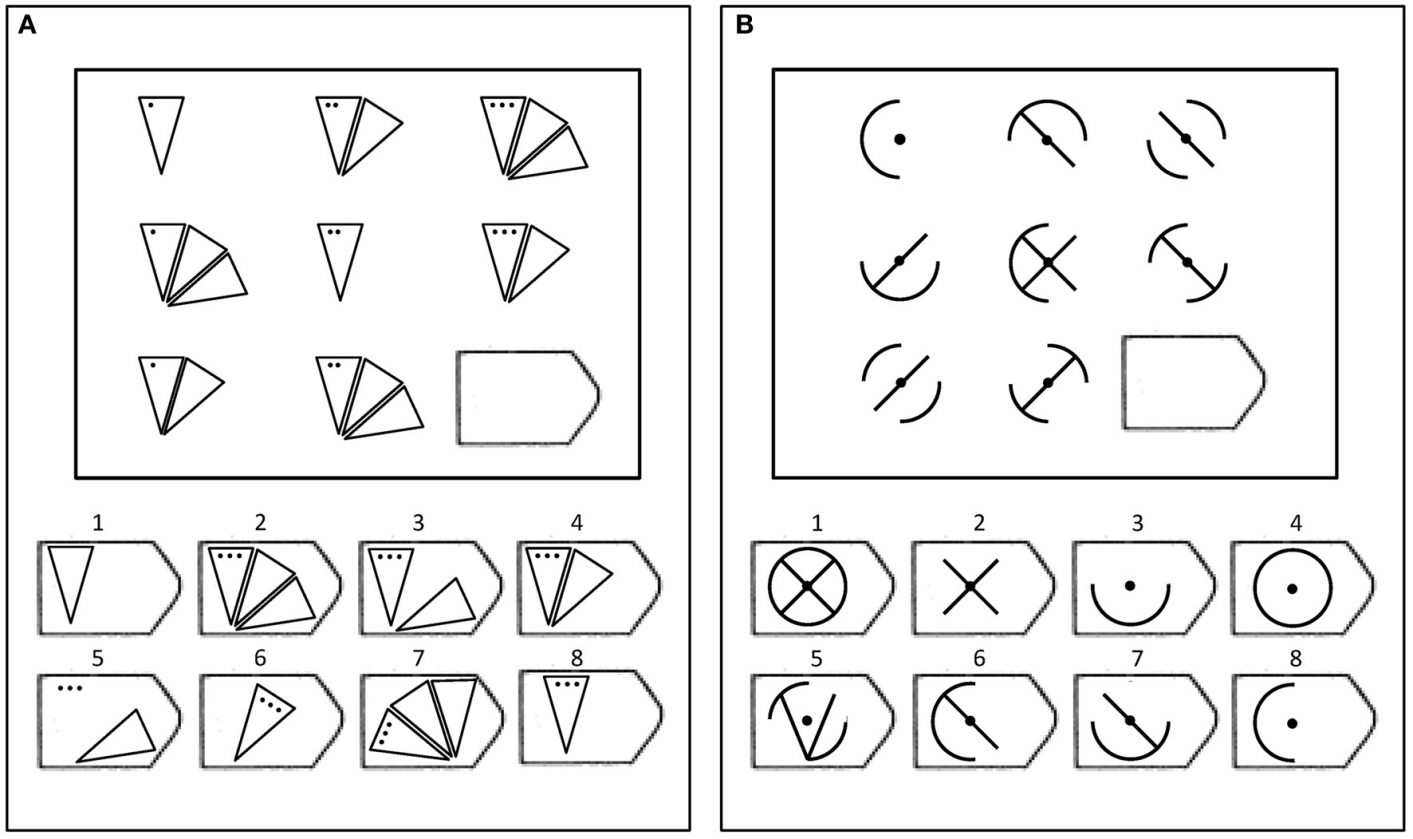
**Two examples of matrices like those in the Raven's test**. **(A)** Example of an item containing a pairwise incremental rule and a distribution of three permutation rule. **(B)** Example of an item containing a constant rule and a distribution of two (XOR) rule.

Carpenter et al.'s (1990) model assumes that people apply one or more rules from a taxonomy of rule types to solve each Raven's problem. To illustrate, Figure 1 presents two sample Raven's-like problems created using different rules. The matrix in panel A contains a *pairwise* incremental rule (i.e., the dots increase across items from left to right) and a distribution of 3, *Dis3*, permutation rule (i.e., objects with 1, 2, and 3 triangles are permuted across rows and columns). The matrix in panel B contains a *constant* rule (i.e., the center dot appears in all items) and a distribution of 2 (logical XOR or *Dis2*) rule (i.e., features which appear in the first two objects do not appear in the third object and features which appear only in one of the first two objects also appear in the third object). Carpenter et al.'s rule taxonomy also included pairwise feature decrements between objects, logical disjunction rules (OR or *addition*) and logical conjunction rules (AND or *subtraction*). Participants must infer these rules from the objects in the matrix and then predict and select the missing lower right object in the matrix from the set of possible response options.

In addition to rule type, problems in Raven's also vary in the number of rule *tokens* or combinations of single rules needed to successfully solve the problem. Difficult problems are characterized by the use of logical operations (e.g., XOR rules) and multiple rule tokens. For example, the matrices in panels A and B (see Figure 1) are typical of easy and difficult Raven's items, respectively. According to Carpenter et al.'s (1990) analysis, the matrix in Panel A contains three rule tokens (i.e., the position of the different numbers of triangles and the number of dots vary across the rows, and the number of dots is constant down the columns), and the matrix in panel B contains six rule tokens (e.g., the four arcs comprising the circle and the two diagonal internal lines). In Carpenter et al. (1990), the total number of rule tokens in a problem explained 57% of the variance in the accuracy rates, and according to their analysis, problems containing distribution of 2 (XOR) rules only appear at the end of the Raven's test where accuracy is the lowest.

Carpenter et al. (1990) compared two production system models that demonstrated the importance of the number and type of rules, and WMC. Both of the models operated by finding correspondences between the symbolically-coded features of the items, transferring these correspondences to a working memory buffer where any rule satisfied by the extracted correspondences was invoked, using the instantiated rules to generate the missing item, and finally, searching through the response options to find the best match. One model (called FAIRAVEN) had no strategic memory organization and did not have access to distribution of 2 (XOR) rules; the other model (called BETTERAVEN) was endowed with better control processes and contained access to all of the rules types. The principles and assumptions used in the development of FAIRAVEN and BETTERAVEN were consonant with observed accuracy, response time, and eye fixation data and the models were able to explain the performance of median Raven's performers and the very best Raven's performers, respectively.

If we assume that increased WMC allows for an improved ability to maintain goals and retain intermediate results and rules necessary to successfully solve the most difficult Raven's items, the implication of the modeling is that performance on more difficult items should be more highly correlated with WMC. However, in subsequent tests of that hypothesis, several studies examined the correlation between WMC and Raven's performance across ordinal item position. Because Raven's is designed such that the items increase in difficulty with order of presentation, the ordinal item position acted as a proxy for item difficulty in these studies (and in our present study). Contrary to expectation, those studies uniformly found that the role of WMC remained invariant across ordinal item position. For example, Wiley et al. (2011) correlated performance on a single measure of WMC with performance on each of the 36 items of the Advanced version of Raven's test (RAPM; Raven et al., 1998) and found that this correlation remained invariant across items^1^. Wiley et al. used an operation span task (OSPAN from here on) to measure WMC. In the OSPAN task, people are presented with a list of memoranda (e.g., letters) for immediate serial recall, but study items are separated by one or more arithmetic equations (e.g., “3 + 5 = 7”) that participants have to evaluate and verify for correctness. Complex span tasks such as OSPAN are a favored assay of WMC because they combine simple memory storage with the simultaneous processing demands that are characteristic of working memory.

**Figure 2:**
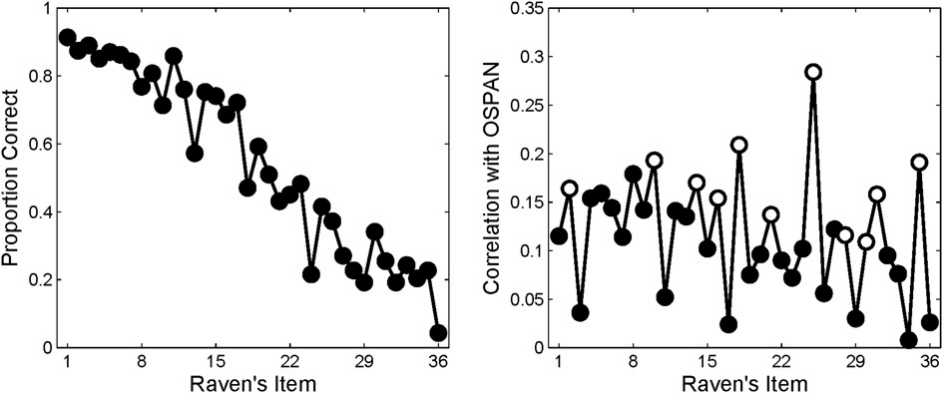
**Observed accuracy (left panel) and observed item-wise point bi-serial correlations (right panel) in Wiley et al. (2011)**. White circles indicate items containing “novel” rule combinations. Right panel adapted from Wiley et al. (2011).

The right-hand panel of Figure 2 shows the item-wise correlations between OSPAN performance and performance on each Raven's item observed by Wiley et al. (2011); the accuracy for each item is shown in the left-hand panel. (The white dots in the right panel index items comprised of novel rule combinations and are discussed further below). Although the pattern is quite noisy, it suggests that there is no systematic relationship between ordinal item difficulty (on the abscissa) and the correlation between performance on those items and WMC (as measured by OSPAN). This impression of an invariant relationship was statistically supported by the failure to find an increasing correlation between OSPAN and the proportion correct within each quartile of the Raven's test.

Similar results have been presented elsewhere (Salthouse, 1993; Unsworth and Engle, 2005). Those reports of invariant item-wise correlations have been used to reject the model of Carpenter et al. (1990), or indeed any other proposal that cites the ability to hold rules and goals in working memory as underlying Raven's performance. The failure to find a selective involvement of WMC has motivated alternative theorizing about the relationship between the Raven's test and WMC.

For example, Wiley et al. (2011) examined whether, irrespective of item difficulty, items that demanded novel rule combinations might require greater working memory involvement. Recall that the items in Raven's are created using a limited number of rules which are thus necessarily repeated across the test. If people learn the rules that appear early in the Raven's tests, then these rules may interfere with rule induction when novel rules are introduced. Consequently, Wiley et al. (2011) hypothesized that the item-wise correlations between Raven's and WMC should be highest for items comprised of novel rules or novel rule combinations (i.e., items 2, 10, 14, 16, 18, 21, 25, 28, 30, 31, and 35 shown with white circles in Figure 2). In support, using OSPAN as a measure of WMC, Wiley et al. revealed the highest item correlations for items requiring novel rules, which appear throughout the Raven's test (see Figure 2, right panel). On this view, it is the novelty of a rule in the sequence of items that triggers a greater involvement of WMC, but not the difficulty of that rule *per se*. A related view holds that the variance shared by WMC and Raven's reflects attentional control mechanisms, which are thought to be uniformly important across all of the Raven's items (Unsworth and Engle, 2005). There is empirical support from other domains that working memory underwrites an ability to filter out distracting information (Conway et al., 2001; Kane et al., 2001; Vogel et al., 2005).

The current state of affairs thus presents a conceptual puzzle: On the one hand, intuition and at least one theory (Carpenter et al., 1990) suggest that the importance of WMC should be accentuated for the more difficult Raven's items, for the simple reason that the easiest items are—by design—solvable by most participants and hence ought not to correlate much with WMC. Such a result would be consistent with a model which assumes that the easiest items incorporate relatively few rule tokens and are solvable using rules available to all participants (e.g., Carpenter et al., 1990).

On the other hand, the invariant item-wise correlation observed by Wiley et al. (2011) is consonant with an attentional view of working memory but runs counter to the model of Carpenter et al. (1990). However, there are several reasons to examine those reports further: First, the counter-intuitive nature of those results deserves to be underscored—after all, how can the correlation between WMC and performance be identical for items that are solved by 90% and 10%, respectively, of participants? Second, and perhaps most important in the present context, the acceptance of an invariant relationship between Raven's performance and WMC may have been premature. It must be recognized that the invariant relationship reflects a failure to reject the null hypothesis, and the “noisiness” of the data is considerable (see right-hand panel of Figure 2). Moreover, existing studies that produced an invariant item-wise correlation were limited by the fact that only a single task (OSPAN) was used to measure WMC—consequently, measurement error or “method variance” from that single task might have masked a relationship between WMC and the more difficult Raven's items in Wiley et al. (2011). In support of this claim, the correlation reported in that paper (*r* ≅ 0.33) falls on the lower end of the range of correlations between WMC and Raven's identified in a recent meta-analysis (i.e., 0.312–0.641; Ackerman et al., 2005). Finally, the analysis of point-biserial correlations across items is problematic due to the necessary heterogeneity that arises as accuracy decreases across the Raven's test.

In summary, we suggest that there are well-supported reasons to suspect that the involvement of WMC in performance actually increases across item difficulty in the Raven's test. Although data to the contrary have been reported, those null effects are based on seemingly noisy data and on limited measures of WMC. We therefore suggest that the issue of how working memory relates to Raven's performance is best considered unresolved at present.

In the remainder of this article, we revisit this issue and resolve it by presenting a simulation and a behavioral study that converge on the conclusion that the role of WMC increases with item difficulty—as predicted by Carpenter et al. (1990) and contrary to the null results reported to date. We first present a simulation study which shows that item-specific correlations can be constant across difficulty levels only if the absolute magnitude of the overall correlation (i.e., across all items) between WMC and Raven's is fairly low: As the overall correlation between WMC and Raven's performance increases, the item-specific correlations can no longer be constant but must also increase across item difficulty. This is a necessary consequence of near ceiling performance on the early items which declines as the items become more difficult combined with a high overall correlation between Raven's and WMC. Only participants with higher Raven's scores (and by implication of the high overall correlation, higher WMC scores) will have correct responses toward the end of the test where only a small percentage of participants respond correctly. We then present a study that related WMC to Raven's performance. Unlike relevant precedents, this study used multiple measures of WMC, thus yielding a composite latent variable less prone to measurement error which was therefore expected to correlate more highly with Raven's performance (cf. Ackerman et al., 2005).

To foreshadow our results, as predicted from our simulations, the behavioral study showed an increase in item-wise correlations with WMC across item difficulty, contrary to the results reported to date (we report a further study in the Supplementary Material that replicated this basic finding). We conclude that the more difficult Raven's items indeed tax WMC more than the easier items, resulting in an increasing correlation with item difficulty that escapes detection only when the overall relationship between Raven's performance and measures of WMC is low. We buttress our conclusions by using a randomization bootstrap of the data to show that the emergence of an item-wise increase in correlation was a necessary consequence of an increasing overall correlation between WMC and Raven's performance.

## Simulation study: the relationship between WMC and raven's performance

In this simulation, we aimed to elucidate the relationship between the overall correlation, *ρ*, between WMC and Raven's and the item-wise point-biserial correlations. We systematically increased the simulated overall correlation and examined the effect on the item-wise correlation. If there is an invariant relationship between WMC and Raven's across items, then the simulated item-wise correlations should not change with Raven's item difficulty irrespective of the overall correlation. By contrast, if difficult Raven's items necessitate more WM, then there should be greater correlations between WMC and the more difficult Raven's items than between WMC and easier items. To maintain parity with previous results, the simulations used the means, standard deviation, minimum and maximum values and observed proportion correct for each item reported by Wiley et al. (2011) for the OSPAN and RAPM tests. We first illustrate that the *ρ* reported by Wiley et al. (2011) does, in fact, lead to an invariant item-wise relationship between WMC and Raven's. We then illustrate that as *ρ* increases, the item-wise correlations increase with increasing item difficulty. That is, the slope of the item-wise correlation function increases as *ρ* increases (see Figure 4).

### Method

To capture the relationship between WMC and Raven's on an item-by-item basis, we made the following assumptions: First, the entire sample of Raven's scores and WMC scores were generated from a bivariate normal distribution in which each point in the distribution represented a single participant's overall Raven's and WMC scores. The bivariate normal distribution had means (OSPAN = 0.61, RAPM = 0.55), standard deviations (OSPAN = 0.15, RAPM = 0.16), and population correlation (*ρ* = 0.3) that mirrored the values reported by Wiley et al. (2011). For each simulation replication, 255 synthetic subjects were sampled from this joint distribution, with the data truncated to fall within the observed range to match Wiley et al.'s 2011 results as closely as possible.

**Figure 3:**
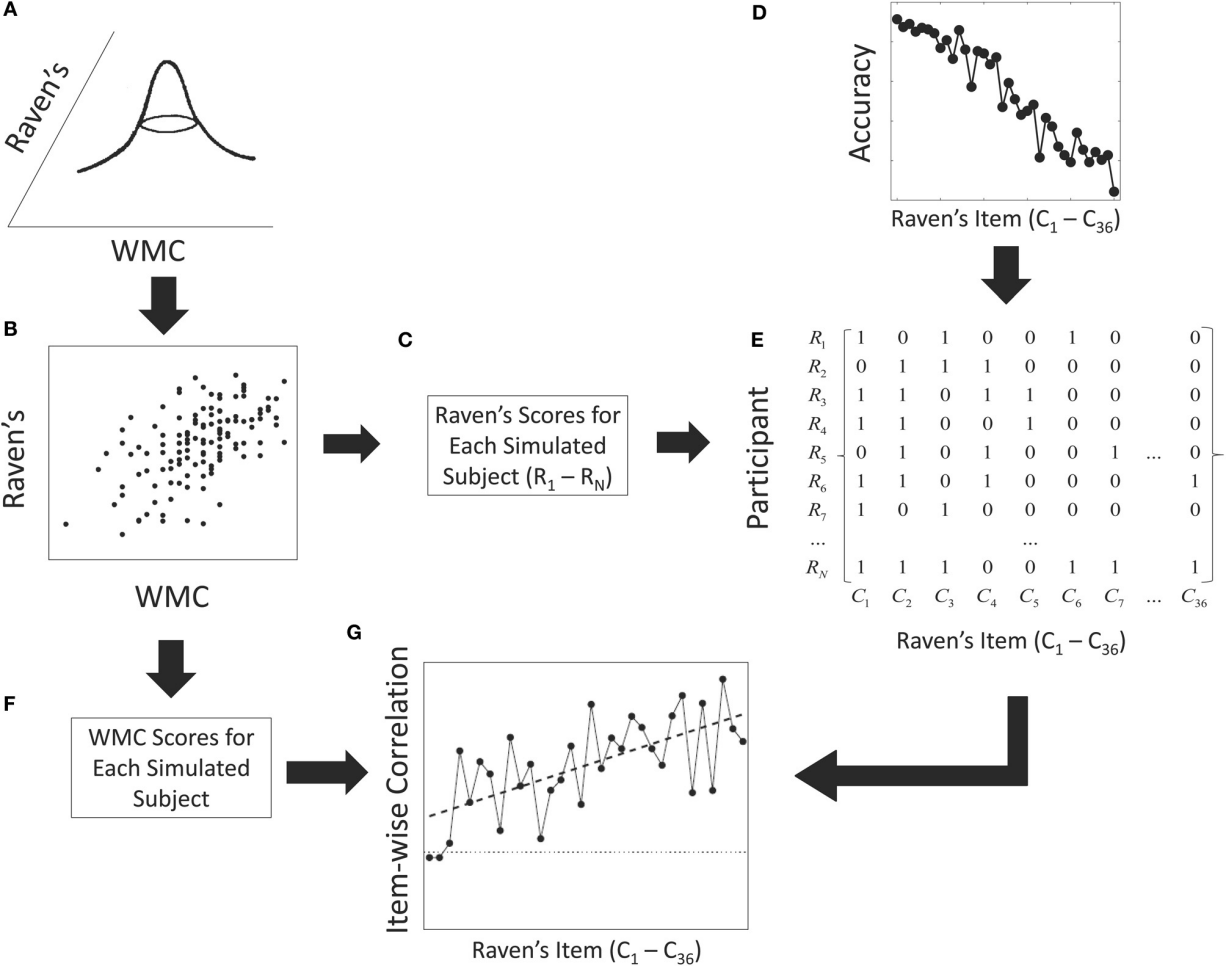
**Data generation process used in the simulation study**. First, a pair of individual WMC and Raven's scores are sampled from a bivariate normal distribution **(A)**. The sample shown in panel **(B)** is from the current behavioral study. The Raven's scores for each simulated subject **(C)** and the accuracy on each Raven's item **(D)** are used to constrain the generation of a simulated binary vector of correct and incorrect responses for each simulated subject. See text for details. The columns of the simulated binary matrix **(E)** are correlated with the overall WMC scores for each simulated subject **(F)** to produce an item-wise correlation function **(G)**.

Across Raven's items, accuracy is by design highest for items which appear early on the Raven's test and then decreases rapidly with ordinal item position (left panel of Figure 2). This result was embodied in our simulation by satisfying the following constraints: First, for each synthetic participant we generated a vector representing item-wise performance where each item could either be correct {1} or incorrect {0}. For each simulation replication, a binary {0,1} matrix was then constructed with the items represented in columns and each subject summarized by a row that represented item-by-item responses (see Figure 3E). The matrix was generated using a sequential Monte Carlo method (Chen et al., 2005) to satisfy the constraints that rows had to sum to the sampled RAPM score for each participant and that columns had to sum to the observed overall performance on each item. Thus, the sums within each column followed the exact pattern shown in the left panel of Figure 2 (replicated in panel **D**; Figure 3) and the sums within each row were distributed with the mean and standard deviation of RAPM scores observed by Wiley et al. (2011).

Specifically, starting with the first column of the matrix, a number of participant-cells, equal to the number of participants who responded correctly for that item, were randomly set to unity with probabilities proportional to the sampled overall proportion correct for that given subject (Figure 3B). The total correct for the participant associated with any participant-cells set to unity was decreased by 1, and the sampling scheme was repeated on the next column and so on. That is, each synthetic participant starts with total number correct as sampled from the joint distribution between Raven's and WMC (Figure 3A). On each step of the sampling process, the probability of any participant making a correct response is determined by the total overall correct for that subject (panel **C**) and overall proportion correct for that item (panel **D**). By definition, items with higher accuracies are responded to correctly by more participants. After sampling the correct responses for an item, any participant who responded correctly to that item has the total correct score decremented by one reflecting the number of correct responses remaining to be allocated for that participant. Sampling in this manner results in random binary matrices with specific row and column sums (panel **E**; cf. Ryser, 1963). This data-generating model thus satisfied the two constraints just noted; namely, item-specific accuracy and inter-individual variability.

Finally, point bi-serial correlations were computed between the generated WMC scores for each subject (Figure 3F) and each column of the simulated binary matrices to produce an item-wise correlation function (panel **G**). It is important to note that only the Raven's scores for each participant and the overall accuracy on the Raven's test contribute to the generation of the binary matrices. The only link between WMC and Raven's is through the population correlation assumed in the bivariate normal distribution.

Across 10,000 simulation replications, WMC scores and RAPM scores were sampled anew for each simulated participant according to a specified population correlation with the total RAPM score, each time drawing a new set of data from the bivariate normal distribution. We examined the relationship between *ρ* and the item-wise correlation function across several simulated conditions in which we increased *ρ* from 0.1 to 0.3, 0.5, 0.7, 0.8, and 0.9 to explore the entire range of positive correlation values.

### Results

**Figure 4:**
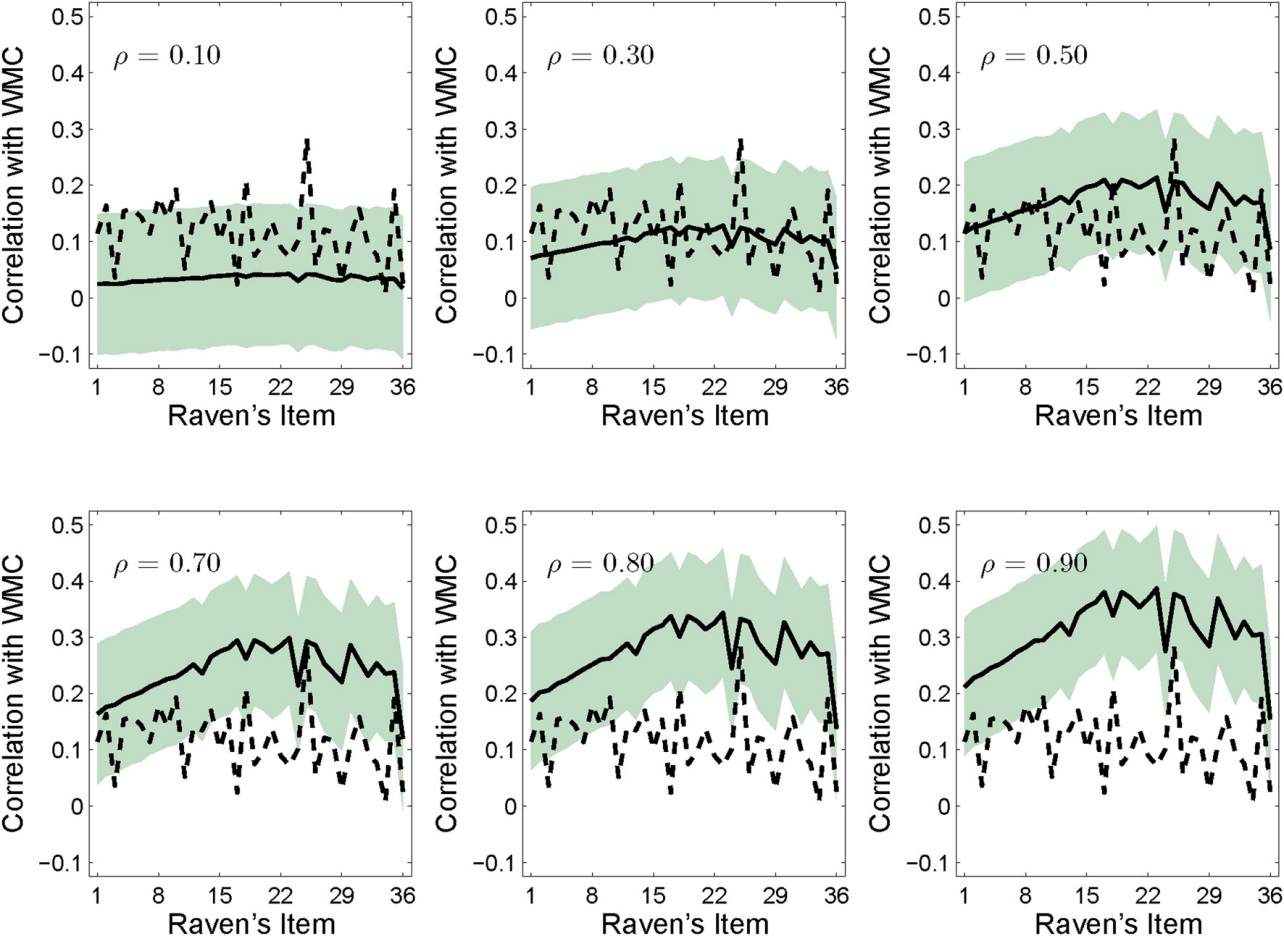
**Simulation results for point bi-serial correlations between WMC and the Raven's items for population correlations of 0.10, 0.30, 0.50, 0.70, 0.80, and 0.90, respectively**. Simulations involved 10,000 replications each, and the shaded areas represent the 95% confidence regions for the mean simulated correlations (the solid line). The upper center panel matches the overall correlation and item-wise results in Wiley et al. (2011) shown by the dotted line.

As shown in the upper middle panel of Figure 4, with *ρ* = 0.3, the simulation reproduced the point-biserial item-wise correlations observed by Wiley et al. (2011); the 95% confidence region of the simulation (computed by finding 1.96 times the standard deviation of the point bi-serial correlations for each item across all 10,000 simulated replications) comfortably straddled the observed values. This simulation result confirmed that our data-generating model was able to capture the basic results observed by Wiley et al. (2011) at the level of overall item accuracy and item-wise correlations with WMC.

Now consider the pattern across levels of *ρ* shown in Figure 4: When *ρ* was moderate or large, the influence of WMC increased with item difficulty—in line with the model of Carpenter et al. (1990) and contrary to previously reported invariant item-wise correlations. The slopes of the simulated item-wise correlations were 0.0002, 0.0006, 0.0009, 0.0013, 0.0015, and 0.0016 for *ρ* = 0.1, 0.3, 0.5, 0.7, 0.8, and 0.9, respectively.

This result has an intuitive interpretation that arises naturally from consideration of how the point-biserial correlations are related to the overall correlation: as the overall correlation with WMC and Raven's increases, participants with higher WMC scores also tend to have higher Raven's scores (i.e., they answer more Raven's items correctly) and participants with lower WMC tend to have lower Raven's scores. However, the pattern of correlations across all of the items is constrained by the decreasing accuracy across items. High overall accuracy lowers the item-wise correlation for the early items (i.e., the point bi-serial correlation must be near zero if nearly everyone gets the item correct) resulting in an increasing slope across the entire test. For the later more difficult items, the participants who respond correctly have to come from the pool of participants who have higher Raven's scores *and* higher WMC. Consequently, with a high overall correlation between WMC and Raven's, the point-biserial correlation between WMC and the most difficult Raven's items that have the lowest accuracy, must be higher than the point-biserial correlation between WMC and the easiest Raven's items.

By this reasoning, the simulations predict that if the overall correlation between Raven's and WMC is high *and* accuracy on the first few items is high, then not only will the item-wise correlations be highest for items near the end of the test, but the item-wise correlations will be near zero for items near the beginning of the test because, regardless of WMC, everyone gets these items correct. Consequently, with a high overall correlation and high accuracy, the slope of the item-wise correlation function should be positive^2^.

### Discussion

The simulation results suggest that the rather low correlation between OSPAN and RAPM (*r* = 0.33) observed by Wiley et al. (2011) may have obscured a pattern that would have emerged had the overall correlation been greater. It is important to note that this argument can have force only if the correlation between WMC and RAPM can legitimately be expected to be greater: If the true value of that correlation were in the vicinity of the value observed by Wiley et al. (2011), then any argument about the possible effects of a greater overall correlation would be moot. Fortunately, it is known that WMC, when measured more accurately with multiple tasks to permit latent-variable analysis (e.g., Kane et al., 2005), correlates more highly with tests of fluid ability than observed by Wiley et al. (2011).

We now report a study that used multiple tasks to measure WMC, thus reducing the task-specific variance and measurement error that beset a single-task measure such as OSPAN. We expected the correlation between WMC and RAPM performance to be greater than in relevant previous studies, and on the basis of our simulation results, we expected an effect of item difficulty to emerge.

## Behavioral study

In this study, we tested the primary prediction suggested by our simulations: If the overall correlation between WMC and Raven's is increased, does this produce an increasing item-wise correlation? Like (Wiley et al., 2011), we use the Raven's Advanced Progressive Matrices (RAPM); however, we do not rely on a single measure of WMC, but use multiple tasks and derive a composite latent measure of working memory. To increase generality, we replicated this study, which used the RAPM, using a different version of Raven's, the Standard Progressive Matrices (RSPM). The results of this replication are reported in the Supplementary Material.

To foreshadow, the results indicate that with a high overall correlation between WMC and RAPM, the slope of the item-wise correlation function significantly increases as the items become more difficult. We additionally show that this result generalizes to a further item-wise analysis of the number of rule tokens. However, we find no relationship between item novelty and WMC in contrast to Wiley et al. (2011).

### Method

#### Participants

The participants were 130 volunteers (95 females; mean age 21.12) from the University of Western Australia campus community. Participants received either partial course credit for an undergraduate psychology course or $20 for two 1-hour sessions.

#### Procedure

In the first session of the study, participants completed a battery of four WMC tasks from the WMC battery presented by Lewandowsky et al. (2010). The battery of four WMC tasks presented by Lewandowsky et al. (2010) was written in MATLAB with the aid of the Psychophysics toolbox (Brainard, 1997; Pelli, 1997). Full details for these tasks can be found in Lewandowsky et al. (2010), and we survey them only briefly here.

#### Memory updating (MU)

The MU task required participants to (a) store a series of numbers in memory, (b) mentally update these numbers based on a series of arithmetic operations, and (c) recall the updated numbers. On each trial, three to five frames containing random digits were presented on the screen. Following memorization, successive arithmetic operations, (e.g., “+4” or “−3”) were presented in the frames, one at a time for a random number of steps before final recall was cued. The key dependent variable is the proportion of updated digits recalled correctly.

#### Operation span (OSPAN) and sentence span (SS)

On each OSPAN trial, a series of arithmetic equations were presented (e.g., 4 + 3 = 7), each of which was followed by a consonant for memorization. Participants judged the equation for correctness and recalled the consonants immediately after list presentation in the original order. The SS task was identical to the OSPAN, except that instead of judging correctness of an equation, participants judged the meaningfulness of sentences (cf. Daneman and Carpenter, 1980). For OSPAN and SS, the key dependent variable is the proportion of consonants recalled correctly.

#### Spatial short-term memory (SSTM)

The SSTM task was adapted from Oberauer (1993) and involved memorization of the spatial location of circles presented, one-by-one, in various locations in a 10 × 10 grid. Participants used the mouse to indicate the memorized location of the dots in any order by clicking in the corresponding grid cells. For this task, participants are given a score based on how similar their recalled pattern was to the to-be-memorized pattern (see Lewandowsky et al., 2010).

#### Fluid intelligence tests (RAPM)

In the second session, participants completed Sets I and II of the 1962 Raven's Advanced Progressive Matrices. As recommended by Raven et al. (1998), RAPM Set I was included to familiarize participants with the matrices. Participants had 5 min to complete the 12 items in Set I before being given the standard 40 min to complete the 36 items in Set II. We only report the results for the last 36 items (Set II).

### Results and discussion

**Figure 5:**
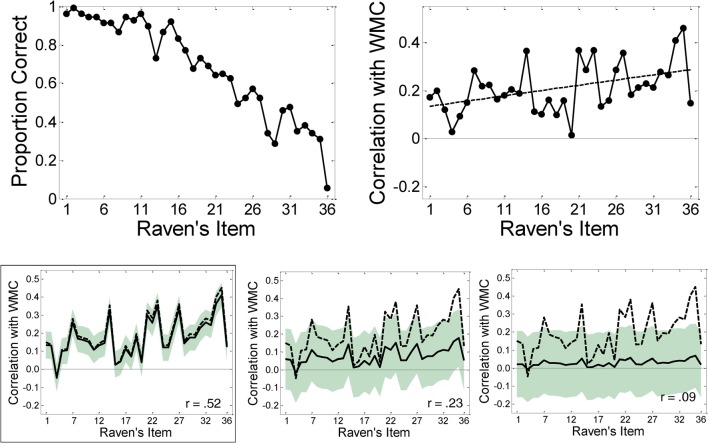
**Top left:** Performance on Raven's advanced progressive matrices items. **Top right:** Observed correlations between working memory capacity (*z*WMC; based on a battery of four tasks) and performance on each Raven's item. The solid line represents the best-fitting regression line (intercept 0.13, slope 0.005). **Bottom panels:** Results from a bootstrapping analysis resulting in correlations of 0.52, 0.23, and 0.09, respectively, between WMC and overall Raven's performance. All bootstrap results are based on 10,000 replications and the shaded areas represent the 95% confidence regions for the bootstrapped means. The framed bottom-left panel matches the overall correlation and item-wise results in the top right panel.

Data from two participants who failed to complete all tasks were removed from the analysis, and data from two further participants were removed for having WMC and Raven's scores less than three standards deviations below the mean, respectively. The final analyses thus used a sample size of *N* = 126. Descriptive statistics for the four WMC tasks and RAPM are shown in Table 1. The top left panel of Figure 5 shows average performance on the RAPM items from Set II. The pattern conformed to expectation in that accuracy decreased with ordinal item position.

**Table 1:** **Means, standard deviations, skewness, and kurtosis for the operation span task (OSPAN), sentence span task (SS), spatial short-term memory task (SSTM), memory updating task (MU), and Raven's Advanced Progressive Matrices (RAPM)**.

**Measure**	***M***	***SD***	**Skewness**	**Kurtosis**
OSPAN	0.71	0.14	−0.99	4.07
SS	0.70	0.15	−0.70	3.30
SSTM	0.84	0.06	−0.14	2.37
MU	0.66	0.18	−0.34	2.48
RAPM	24.47	5.37	−0.34	2.90

For the correlational analyses, we computed a composite measure of WMC by first converting each participant's score on each WM task into a *z*-score, and then computing that person's average *z*-score across the four tasks (*z*WMC). The overall correlation between *z*WMC and the total RAPM score was moderately large, *r* = 0.56, *p* < 0.001.

The top right panel of Figure 5 shows the point bi-serial correlations between WMC and performance broken down across Raven's items, together with the best-fitting regression line. The slope of the regression line (0.004) was significantly greater than zero, *t*_(34)_ = 2.87, *p* < 0.01, *r*^2^ = 0.20. This slope is greater than the slopes predicted from our simulation because overall accuracy across all of the items is higher in our study than in Wiley et al. (2011). Due to the near ceiling accuracy for the first few items in our study, the item-wise correlation for these items is closer to zero, which consequently results in an increased slope. Our data confirm that when there is at least a moderate correlation between WMC and Raven's performance, the item-wise correlations increase with item difficulty, exactly as expected from the simulation results. As shown in the Supplementary Material, we also replicate this effect using a different Raven's test (i.e., the Standard Progressive Matrices) and a different sample of participants.

#### Operation span

To provide further empirical confirmation that a reduction in the overall correlation between WMC and RAPM attenuates the item-wise effect, we examined the correlation between the OSPAN subtask and RAPM. For this task, the overall correlation with Raven's was much lower, *r* = 0.36, *p* < 0.001. The slope of the regression line was not significantly greater than zero, *t*_(34)_ = 1.39, *p* > 0.05, *r*^2^ = 0.05. This result replicates the null slope found by Wiley et al. (2011), and is explained by the low overall correlation between OSPAN and Raven's as predicted by our simulations.

#### Multilevel analysis

To further analyze the relationship between *z*WMC, item difficulty and novelty, and performance on Raven's, we conducted a multilevel logistic regression (Gelman and Hill, 2007), which circumvents problems due to items with very high or very low accuracy by relying on the logistic (or inverse-logit) function to model the accuracy proportions for each item. There are a number of different possible models based on various combinations of WMC, ordinal item position (as a proxy for difficulty), item novelty (cf. Wiley et al., 2011), and their various interactions that might explain the RAPM accuracy data; however, given our theoretical focus on the effects of item difficulty and the results of the simulation study, we examined only two models in detail: The first model includes WMC, ordinal item position, and the interaction between these variables. The second model includes these three effects plus the effect of item novelty. For both models, we systematically tested alternative random-effect models (i.e., letting one or more of intercept, ordinal item position, and novelty vary randomly across participants) and determined the “best” model using BIC.

The logistic regression assumes that the predictors are linearly related to the logit transformation of the dependent variable; consequently, we examined the relationship between each variable and accuracy using a White test for non-linearity (Lee et al., 1993). Ordinal item position showed a demonstrable non-linear relationship with accuracy χ^2^ (2) = 61.12, *p* < 0.001. A Box–Tidwell analysis indicated that the non-linearity could be removed by raising ordinal item position to a power of 1.704, χ^2^(2) = 4.29, *p* = 0.12 (see Box and Tidwell, 1962). None of the other variables showed any non-linear relationship with the largest χ^2^ being for *z*WMC (χ^2^(2) = 2.86, *p* = 0.24).

Exponentiating ordinal item position to correct for non-linearity, our first model is given by the following equation:

(1) $$y_{ij}=\beta_{0}+\beta_{z}zWMC_{i}+\beta_{N}N_{j}^{\gamma }+\beta_{z\times N}zWMC_{i}\times N_{j}^{\gamma }+\left(S_{0}+e_{ij}\right)$$

where *y*_*ij*_ is a binary response variable indicating whether participant *i* made a correct (1) or incorrect (0) response on item *j*, *z*_*i*_ is the *z*WMC score for participant *i*, ψ_*j*_ is the ordinal item position of item *j*, λ equals 1.704 (as indicated by the above Box–Tidwell analysis), *S*_*i*_ is the set of subject random effects and *e*_*ij*_ is an error term. The linearization of the item effect has no bearing on our interpretation of the results as item position is only an ordinal proxy for difficulty. Consequently, regardless of how that variable is transformed it retains the ordinal association with the unknown scale of actual difficulty.

We tested this model using only the intercept as a random effect (e.g., Model 1, see Table 2), the intercept plus ψ^λ^ as random (Model 2), and intercept, ψ^λ^ and novelty all as random (Model 3). Comparison of the BICs pointed to the model in which only the intercept varied randomly as being preferable (i.e., Model 1). This model revealed significant effects of *z*WMC (*p* < 0.001), ordinal item position (ψ^λ^, *p* < 0.001), and the critical *z*WMC × ordinal item position interaction (*p* < 0.01). The latter interaction confirms that WMC played an increasingly important role as item difficulty increased, precisely paralleling our initial correlation-slope analysis^3^.

**Table 2:** **Estimated parameters (and standard errors) of mixed effects modeling of the RAPM behavioral study**.

**Parameters**	**Model 1**	**Model 2**	**Model 3**	**Model 4**
**FIXED**				
Intercept (*β*_0_)	**2.93 (0.11)**	**2.98 (0.12)**	**2.98 (0.12)**	**2.91 (0.11)**
zWMC (*β*_*z*_)	**0.53 (0.14)**	**0.53 (0.15)**	**0.53 (0.15)**	**0.52 (0.14)**
Ordinal item position (*β*_*ψ*_)	**−0.01 (0.0003)**	**−0.01 (0.0004)**	**−0.01 (0.0005)**	**−0.01 (0.0003)**
zWMC × Ordinal item position (*β*_*z* × *ψ*_)	**0.001 (0.0005)**	**0.001 (0.0005)**	**0.001 (0.0005)**	**0.001 (0.0005)**
Novelty *β*_*κ*_				0.08 (0.09)
**RANDOM**				
Intercept *s*_0_	0.67 (0.59)	−0.05 (0.06)	−0.06 (0.06)	−0.14 (0.06)
Ordinal item position *s*_*ψ*_		0.0001 (0.0001)	0.0001 (0.0001)	
Novelty *s*_*κ*_			0.003 (0.006)	
**EVALUATION**				
df	5	7	10	6
BIC	4089	4097	4121	4097

All significant coefficients are in bold font. 0 = intercept, *z* = *z*WMC, ψ = ordinal item position, κ = item novelty, *df* = degrees of freedom, *BIC* = Bayesian Information Criterion.

To test whether item novelty affected accuracy, we added the rule novelty of each item (*κ*, as defined in Wiley et al., 2011) as a fixed factor as follows:

(2) $$y_{ij}=\beta_{0}+\beta_{z}z_{i}+\beta_{\psi }\psi_{j}^{\lambda }+\beta_{\left(z\times \psi \right)}z_{i}\times \psi_{j}^{\lambda }+\beta_{\kappa }\kappa +\left(S_{i}+e_{ij}\right)$$

As shown in Table 2 (see Model 4), the novelty effect was not significant (*p* = 0.39). Contrary to Wiley et al.'s (2011) conjecture, increased WMC was not related to better performance on items with novel rule combinations. Indeed, this is evident if once compares the item-wise correlations from our study (see Figure 5) with the item-wise correlations shown in Figure 2. This is perhaps unsurprising given that others have also failed to replicate the novelty effect (Harrison et al., 2011).

Having once more confirmed the principal result predicted from our simulation, we now turn to several other analyses which further elucidate the relationship between WMC and Raven's.

#### Bootstrapping analysis

Our simulation study began from the overall correlation between Raven's and WMC and simulated binary matrices to examine what happened to the simulated item-wise correlation function when the overall correlation was increased or decreased. Having collected data that confirmed the expectations of our simulation, we next conducted a bootstrapping analysis in which we begin from the *observed* binary matrices and then simulate the effect of decreasing the overall correlation to examine the resulting effect on the item-wise correlation function.

In other words, to confirm that the magnitude of the overall correlation was responsible for the emergence of an item-difficulty effect in our study, we conducted bootstrapping analyses based on the observed subject × item (126 × 36) response matrix (with rows ordered according to the observed *z*WMC). Specifically, the overall correlation between *z*WMC and Raven's was gradually reduced by generating new *z*WMC scores for each participant and examining the effect of that manipulation on the item-wise correlations. In terms of the our simulation reported at the outset, we were effectively replacing the simulated binary matrices (Figure 3E) with our observed binary matrices and reducing the overall correlation between WMC and Raven's while maintaining observed accuracy for each item and the observed Raven's scores for each participant. Thus, the bootstrapping analysis can be thought of as the converse of the simulation procedure.

We created three conditions, each involving 10,000 bootstrapping runs. For each run, a new vector of *z*WMC scores was randomly derived from the observed values according to: *zWMC*^*n*^ = *ν* × *zWMC* + ϵ where ϵ $\sim N\left(0,\sqrt{\left(1-\nu^{2}\right)}\right)$ and *ν* varied across conditions. This new vector contained *z*WMC scores which were derived from the observed *z*WMC scores but had a reduced correlation with the observed Raven's scores. The rows of the observed binary response matrix were then re-sorted according to the new vector *z*WMC^*n*^ yielding another bootstrapped replication with a specified correlation between *z*WMC and RAPM that maintained the overall item-wise error rate and overall Raven's correct for each participant observed in the study. Item-wise correlations were then computed between the bootstrapped replication and the actual *z*WMC scores.

The three bootstrapping analyses used ν = 0.95, 0.50, and 0.20, respectively, which yielded actual correlations *z*WMC × RAPM of 0.53, 0.23, and 0.09 (left, center, and right panel in bottom row of Figure 5, respectively). These actual correlations span a large range of possible overall correlations between WMC and Raven's. The bottom left panel provides an idea of the variability expected when the population correlation is approximately equal to that observed in the study. The remaining two panels show that as the population correlation decreases, so does the slope of the item-wise correlations. The center panel roughly corresponds to the correlation observed by Wiley et al. (2011) and confirms that the effect of item-difficulty is sufficiently small under those circumstances to escape statistical detection when statistical power is insufficient.

#### Rule token analyses

We also examined whether the increasing item-wise correlations held corollary implications for a pattern of effects based on Carpenter et al. (1990)'s classification of the rules associated with the different Raven's problems. Recall that Carpenter et al. accounted for differences in Raven's performance by assuming differential access to different types of rules (e.g., pairwise, Dis3, Dis2, and Addition and Subtraction) and the ability to manipulate different numbers of rule tokens. People who perform poorly at Raven's were hypothesized to have less capacity for the manipulation and storage of rule tokens.

In contrast to the expectation that WMC should be correlated more highly with problems requiring more rule “tokens” to solve, Wiley et al. (2011) found that the correlation between WMC and performance did not increase with an increasing number of tokens (*r* = 0.25, 0.24, 0.33, and 0.21, for items involving 1, 2, 3, and 4 tokens, respectively). To examine the relationship between rule tokens and WMC in the current study, we conducted a multilevel logistic regression examining the interaction between WMC and the number of rule tokens using the following model:

(3) $$y_{ij}=\beta_{0}+\beta_{z}z_{i}+\beta_{\psi }\psi_{j}^{\lambda }+\beta_{\left(z\times \psi \right)}z_{i}\times \psi_{j}^{\lambda }+\left(S_{i}+e_{ij}\right)$$

where *β*_*N*_*N*^γ^_*j*_ is the number of rule tokens for each item *j* raised to the power of *γ* = 2.32, which is the exponent returned by the Box–Tidwell analysis to correct a non-linear relationship between the number of rule tokens and logit-transformed accuracy (χ^2^ = 19.79, *p* < 0.001). No other variables showed a significant non-linear relationship. Finally, we compared a model which included only the intercept as a random effect against a second model which included both the intercept and number of tokens as random effects; the BICs were 3118 and 3131, respectively. Consequently, we only report the results of the intercept-only random effects model.

This analysis necessarily relies on a subset of the data because Carpenter et al.'s (1990) derivation of the number of rule tokens excluded several items for technical reasons. Our analysis revealed a significant negative coefficient for the number of tokens [standard error shown in parentheses, *β*_*N*_ = −0.10 (0.005), *p* < 0.001], indicating that accuracy decreases with the number of tokens. More importantly, there was a significant coefficient for the *z*WMC × number of tokens interaction [*β*_*z*×*N*_ = 0.02 (0.007), *p* < 0.01], which further qualified the individual contribution of WMC [*β*_*z*_ = 0.54 (0.12), *p* < 0.001]. The intercept coefficient was also significant [*β*_*N*_ = 2.08(0.09), *p* < 0.001]. This indicates that higher WMC helps you more when there are more tokens, which is in accord with our previous analyses and with Carpenter et al.'s (1990) theoretical proposal.

## General discussion

The simulation results and behavioral data converge on the same conclusion: When there is a moderate to strong overall correlation between WMC and performance on the Raven's test of fluid abilities, then the role of WMC becomes increasingly more important as item difficulty increases. The simulation reported at the outset demonstrated that under simple yet reasonable data generating assumptions, the increasing item-wise correlation is inevitable if the overall correlation is high. This prediction was confirmed using a behavioral study (and replicated using a different measure of Raven's, see Supplementary material). Why, then, have previous experiments investigating this issue failed to detect this modulating role of item-difficulty?

### Relationship to previous findings

Our simulation, multilevel logistic regression, and the bootstrapping analyses suggest that other studies failed to find an effect of item difficulty because in their cases the overall correlation involving WMC was insufficient in magnitude. One apparent exception to this analysis involves the results of Salthouse (1993), who also reported a non-significant item-wise slope despite showing a high overall correlation between WMC (measured by OSPAN and SSspan) and Raven's, *r* = 0.59. However, we suggest that this high overall correlation may have been artifactual. Salthouse (1993) tested a large sample of participants from across four age groups (students with a mean age of 20 and adults with ages ranged 20–39, 40–59, and 60–79). The correlation was reported by combining data across all samples, thereby confounding between-group and within-group variability. Indeed, as Salthouse (1993) reported, the average overall Raven's score decreased with the age of the participants. Likewise, WMC was also negatively and highly correlated with age (*r* = −0.54). Consequently, the true correlation between WMC and Raven's performance—that is, the remaining correlation when between-group differences are removed—is likely to have been much lower. In support of our claim, Salthouse (2000)'s reanalysis of Salthouse (1993) revealed unique variance associated with the more difficult items; that is, unique variance remained in the relationship between age and the hardest Raven's problems after controlling for variance in the easiest items. Although not reported at the level of detail necessary to determine whether the itemwise correlation increases with item difficulty, this analysis is consistent with this notion.

It must be underscored that we reported two independent replications of a significant item-difficulty effect using two variants of the Raven's test, each of which subsumed a null (RAPM) or reduced (RSPM, see Supplementary Material) effect of item difficulty when WMC was measured using a less optimal measure of WMC. That is, we replicated existing null results when we did not remove “method variance” from our assay of WMC while simultaneously showing that acceptance of the that null result was ill-advised because it is rejected when a composite measure of WMC is used that at least partially controls for method variance.

Finally, we reiterate that the appropriate analysis for this data is the multilevel level logistic regression. We have explored the item-wise correlation due to the precedents set by Wiley et al. (2011) and Unsworth and Engle (2005) but note that this analysis is inherently problematic due to ceiling effects at the beginning of the test and floor effects at the end of the test. On the basis of those problems, one may be tempted to speculate that the magnitude of the overall correlation only affects the problematic item-wise correlation analysis but not the multilevel modeling. In other words, it may be ok to use a single measure of WMC if one applies the appropriate multilevel logistic analysis. We examined this by rerunning the multilevel regression using our best fitting model (Model 1, see Equation 1), but substituting OSPAN for zWMC. In this analysis, the OSPAN × ordinal item position interaction was not significant (*p* = 0.09) indicating that even with the multilevel regression, the use of a single measure of WMC would hide the underlying relationship between WMC and Ravens.

Lest one wonder why the Raven's test, one of many assays of fluid intelligence, is worthy of study it must be recalled that the Raven's test is a rule induction task *par excellence*; consequently, understanding the relationship between WMC and Raven's should inform theories of the relationship between WMC and other induction tasks, such as category learning (e.g., Lewandowsky, 2011; Craig and Lewandowsky, 2012; Sewell and Lewandowsky, 2012), theories of rule-based categorization (Fific et al., 2010; Nosofsky and Little, 2010; Little et al., 2011, 2013; Little, 2012), and theories of individual differences in categorization (Yang and Lewandowsky, 2004; Little and Lewandowsky, 2009; Sewell and Lewandowsky, 2011). Ultimately, understanding the relationship between Raven's and WMC has implications for how to formalize capacity limitations in complex inferential tasks in theories of human intelligence.

### Toward a computational model of raven's and WMC

Our work presents a novel account of the relationship between WMC and Raven's that simultaneously predicts when one should expect to see a positive item-wise relationship and when that relationship should be absent. On the surface, this may appear to be merely a statistical issue, but given the intense psychological attention and interpretation this issue has received, its resolution has considerable psychological implications. In particular, our research limits reliance on a result which has been a substantial barrier to theorizing in this domain. Previously, any model hoping to account for the relationship between WMC and Raven's also would have to explain the invariant relationship across item difficulty. The present result shows that this is not the case and provides tight constraints on quantitative models of WMC and Raven's. We now know that any model attempting to explain the relationship between the two has to predict that high WMC will allow you to do well on hard items in a manner that increases the slope of the item-wise correlation function with the overall correlation.

Our results are compatible with theoretical analyses of Raven's performance that appeal to working memory as a repository for rules and intermediate results (e.g., Carpenter et al., 1990). Although those theoretical views have fallen out of favor, largely due to the apparent absence of a modulating effect of item difficulty on the relation between WMC and Raven's performance, our results suggest that abandoning those approaches may have been premature.

Although Carpenter et al. (1990) provided a computational theory compatible with the current results, other models may also be able to predict the present pattern (e.g., Rasmussen and Eliasmith, 2011; Little et al., 2012). For one, Verguts et al. (2000)'s suggestion that high performers on Raven's sample rule tokens faster than poor performers on Raven's should also predict that WMC has an increasing influence as the items increase in difficulty. In a fixed period of time, faster sampling would result in a greater number of rule samples. In this model, WMC could be represented by the number of samples that can be held in WM at any one time. A similar proposal was recently suggested by Rasmussen and Eliasmith (2011). If early Raven's items only require a few tokens to solve, then a limited number of samples would suffice, but for more difficult Raven's items, a larger number of samples would increase the probability making a correct response on these items. Such theories highlight the key role of WM in Raven's as a repository for rule tokens or samples and predict an increasing influence of WMC as Raven's items increase in difficulty.

A synthesis of these two accounts is possible by extending a recent Bayesian model of Raven's proposed by Little et al. (2012). The model considers rule induction in Raven's as Bayesian inference in which a set of rules with some prior probability, most likely determined by the relative complexity of each rule, are evaluated based on their ability to have plausibly generated the features of the items shown in the matrix. The rules are then used to predict the missing object in accord with their posterior probability. This model accurately predicts correct and error responses for both RSPM and RAPM. Two natural modifications of this model could potentially link theoretical accounts based on the capacity of working memory (e.g., Carpenter et al., 1990) to accounts based on learning which rules are relevant in a given test (e.g., Verguts et al., 2000). First, rather than assuming a complete representation of the prior probabilities of each rule, an approximate prior could be used in which each rule is represented by a number of samples, proportional to observed WMC. For any given problem, the samples would be updated using importance sampling to form an approximate posterior over the rules (see e.g., Shi et al., 2010).

The second modification is to allow the samples to be updated across items using particle filter sampling (Doucet et al., 2001). In the particle filter model, a set of particles representing possible rules applied to some feature are drawn in proportion to their prior probabilities. Initially, their prior probability is inversely proportional to complexity, but as one progresses through the Raven's items, probabilities are updated in proportion to how successful the rule has been previously. Particles representing rules are maintained if they work for the objects in the first two rows and columns, but are replaced with new samples from the prior if they do not. Using the updated set of particles, the missing object is predicted by applying the rules to the features in the third row and column, combining all features into a predicted object, and finding the object in the response set with the highest predicted probability. This framework not only captures the idea that working memory allows for the storage and manipulation of rules, but also the idea that better performance on Raven's is related to learning the set of rules likely to apply across items. A particle filter model of Raven's would view both Carpenter et al.'s and Verguts and De Beock's accounts as consonant and provide an integration of both ideas. We leave this as a goal for future research.

One further unresolved question not addressed by the current study is what makes a Raven's item difficult? Here, in line with all relevant precendents, we use item order as a proxy for item difficulty. This operational definition has been embedded, by design, in the Raven's test, and we adopted it here because our focus is not aimed at discovering *what* makes an item difficult. We were concerned with the relationship between items of known variation in difficulty and WMC—and the universally accepted operationalization of difficulty as item number was sufficient to resolve this question. However, we regard the analysis and explanation of item difficulty to be an important conceptual issue that requires further thought and is awaiting resolution.

## Conclusion

In summary, the present research elucidates the relationship between WMC, a core construct in human cognition that accounts for 50% of the variance in fluid abilities, and Raven's Progressive Matrices, a paramount inductive test and one of the most popular assays of fluid intelligence. We have demonstrated that higher WMC is associated with better performance on more difficult Raven's items. This relationship is only detectable when the overall relationship between WMC and Raven's is high. Our results provide a new challenge for theories of the relationship between WMC and Raven's: namely, any computational theory must predict the tight coupling between WMC and Raven's overall, and WMC and each of the items on the Raven's test.

### Conflict of interest statement

The authors declare that the research was conducted in the absence of any commercial or financial relationships that could be construed as a potential conflict of interest.

## Footnotes

^1^This invariant relationship was first noted by Unsworth and Engle (2005); however, their study was subtly biased against finding an item-wise effect because of the use of a shorter, non-standard time period for completion of the test (30 instead of 40 min). This non-standard timing made it more likely that performance on the most difficult items would be near the floor (because most people ran out of time before solving those items), thereby necessitating their removal and consequently reducing the power of the analysis. Hereafter, we focus on Wiley et al.'s (2011) result which also showed a null effect but used a standard 40 min presentation time.

^2^Of course, a floor effect for the most difficult items would cause the item-wise correlations to be near zero at the end of the Raven's test, which would result in an inverted U-shaped item-wise function if the overall correlation was high—as we indeed observed in the simulation. However, in Wiley et al. (2011) and in our behavioral study, all of the items excepting the final item had performance well above zero. We recognize that describing the item-wise correlation function as “increasing” is contingent on the absence of a floor effect for the final Raven's items. However, regardless of whether the function is increasing or U-shaped, our interpretation of the relationship between the overall and item-wise correlation is unchanged (i.e., the item-wise correlation is increasingly *less invariant* with an increasing overall correlation).

^3^While the exponentiation demonstrably resolves the non-linearity, it raises the potential concern that exponentiating might add undue extra leverage to the later items on the test. To address this question, we conducted a leverage analysis by removing each (exponentiated) item in turn and determining the extent to which the parameter estimate for the crucial *z*WMC × *ψ*^λ^ interaction changes. This leverage analysis showed that there is no correlation between an item's ordinal position and the magnitude of the effect that its removal has on the estimate of the interaction (*r* = −0.13, *p* = 0.44). In other words, the amount of leverage that each item has does not increase with an item's difficulty. This confirms that our transformation of the independent variable did not inadvertently imbue items of greater difficulty with greater leverage (although it must be noted that even that, by itself, would not create an interaction with WMC; increasing leverage may be a potential problem but in the absence of an explanation that also invokes WMC it is by itself insufficient to generate the interaction.)
