# Supplementary material for: Working memory capacity and fluid abilities: the more difficult the item, the more more is better
Source: Front Psychol. 2014 Mar 21;5:239. doi: 10.3389/fpsyg.2014.00239 (PMC3968765; doi:10.3389/fpsyg.2014.00239)
Supplement: Supplementary file 1 [file Presentation1.PDF]

Appendix

Behavioral Study: Raven's Standard Progressive Matrices

In this study, we replicate the increasing correlation found with the RAPM using the *Standard* version of Raven's (RSPM). The RSPM differs from the RAPM used by Wiley et al. (2011) in terms of number and ordering of test items. Whereas items in RAPM are meant to be ordered by item difficulty, in RSPM items are blocked into clusters using the same rules (e.g., in RSPM, set C contains incremental pairwise transformation rules, set D contains distribution of 3 permutation rules and set E contains additive, subtractive and distribution of 2 XOR rules in the taxonomy introduced by Carpenter et al., 1990).

The RAPM was designed to better discriminate between people with higher abilities (Raven et al., 1998). Despite these differences, the items on both tests require the application of similar rules; consequently, we expect cognitive processing and thus the relationship with WMC to be the same on both tests. Indeed, both tests measure the same construct, a general cognitive attribute termed educative ability (Paul, 1985; Raven et al., 1998, 2000). The RSPM and RAPM are also comparable in their levels of concurrent validity (see Raven et al., 1998, 2000, for details). For example, Burke and Bingham (1969) and Burke (1985) found the RSPM and Wechsler Adult Intelligence Scale (WAIS) to correlate at .66, and .75, respectively, whereas Paul (1985) and Vernon (1983) found the RAPM and WAIS to correlate at .67 and .72 respectively. Likewise, the two versions of Raven's both correlate at similar levels with measures of WMC. Conway, Cowan, Bunting, Therriault, and Minkoff (2002) and Engle, Tuholski, Laughlin, and Conway (1999) found overall scores on the RSPM and OSPAN to correlate at .20 and .34 respectively, compared to correlations involving the overall RAPM score of .37 (Ackerman, Beier, & Boyle, 2002), .43 (Jurden, 1995), and .30 (Kaufman, DeYoung, Gray, Brown, & Mackintosh, 2009).

Method

Participants. The participants were 143 volunteers (94 female; mean age 20.90) from the University of Western Australia campus community. Participants received either partial course credit for an undergraduate psychology course or $30 for three 1-hour sessions involving another unrelated experiment. Here we only report the Raven's and WMC results collected during the first and second sessions, respectively.

Procedure. The first session of the RSPM study was identical to that in the RAPM study. Participants completed a battery of four WMC tasks from the WMC battery presented by Lewandowsky et al. (2010). In the second session, participants completed the 1958 revision of Raven's Standard Progressive Matrices test under standard instructions.

Results and Discussion

Data from 11 participants who failed to complete all tasks were removed from analysis, reducing the final sample size to $N = 132$. Descriptive statistics for the four WMC tasks (using partial-credit scoring; see Lewandowsky et al., 2010) and RSPM are shown in Table A1.

For the remaining analyses, the two easiest RSPM item sets (A and B) were omitted because they are considered practice sets when used with adults (Raven et al., 2000). The top left panel of Figure A1 shows performance for the remaining 36 items (sets C, D, and E), rank ordered by observed difficulty.

For the correlational analyses, we computed a composite measure of WMC by first converting each participant's score on each WM task into a z-score, and then computing that person's average z-score across the four tasks (zWMC). The overall correlation between zWMC and the total RSPM score was $r = .59$. The moderately large correlation, which is nearly twice in magnitude to those typically reported with single tests of WMC (Ackerman et al., 2002; Conway et al., 2002; Engle et al., 1999; Kaufman et al., 2009;

Unsworth & Engle, 2005; Wiley et al., 2011) again attests to the success of using a multi-task battery to measure WMC instead of a single span task.

The top right panel of the figure shows the point bi-serial correlations between WMC and performance broken down across Raven's items, together with the best-fitting regression line. Four items with perfect performance were omitted from the correlational analysis. The slope of the regression line (.009) was significantly greater than zero, $t(30) = 4.73$, $p < .0001$, $r^2 = .43$. The data confirm that when there is at least a moderate correlation between WMC and Raven's performance, the item-wise correlations increase with item difficulty, exactly as expected from the simulation results.

Bootstrapping Analysis. We conducted three bootstrapping analyses of our data based on the observed subject $\times$ item (132 $\times$ 32) response matrix (with rows ordered according to the observed zWMC). The three bootstrapping analyses used $\nu = .95$, .50, and .20, respectively, which yielded actual overall correlations zWMC $\times$ RSPM of .56, .30 and .10 (left, center, and right panel in bottom row of Figure A1, respectively). This analysis confirmed the essential role of the overall correlation between Raven's and WMC in determining the slope of the item-wise correlations in the observed data: reducing the overall correlation decreases the slope of the item-wise correlations.

Operation Span. For comparison with existing results, we examined the correlation between the OSPAN subtask and RSPM which was lower than the correlation involving the composite zWMC, $r = .41, p < .001$. Despite this lower correlation, the slope of the regression line (.007) was still significantly greater than zero, $t(30) = 3.65$, $p < .05$, $r^2 = .31$. This result stands in contrast to the the null slope found by Wiley et al. (2011) using OSPAN; however, it should be noted that the RSPM and OSPAN correlation is larger than the correlation observed in previous reports, and the significant slope is therefore consistent with our simulation results.

Table A1

Means, standard deviations, skewness, and kurtosis for the operation span task (OSPAN), sentence span task (SS), spatial short-term memory task (SSTM), memory updating task (MU), and Raven's Standard Progressive Matrices (RSPM).

Measure	*M*	*SD*	Skewness	Kurtosis
OSPAN	0.71	0.14	-0.96	4.00
SS	0.64	0.19	-0.54	2.93
SSTM	0.86	0.06	-0.44	3.53
MU	0.57	0.18	0.23	2.51
RSPM	51.06	5.31	-0.63	4.06

Figure A1. Top left: Performance on RSPM items in sets C through E. Items are rank-ordered according to observed difficulty (i.e., in decreasing order of performance). Top right: Observed point bi-serial correlations between working memory capacity (zWMC; based on a battery of 4 tasks) and performance on each Raven's item rank-ordered by observed difficulty. Four items with perfect performance were omitted from the correlational analysis. The solid line represents the best-fitting regression line (intercept .088, slope .009). Bottom panels: Results from a bootstrapping analysis resulting in correlations of .56, .30, and .10, respectively, between WMC and overall Raven's performance. All bootstrap results are based on 10,000 replications and the shaded areas represent the 95% confidence regions for the bootstrapped means. The framed bottom-left panel matches the overall correlation and item-wise results in the top right panel.

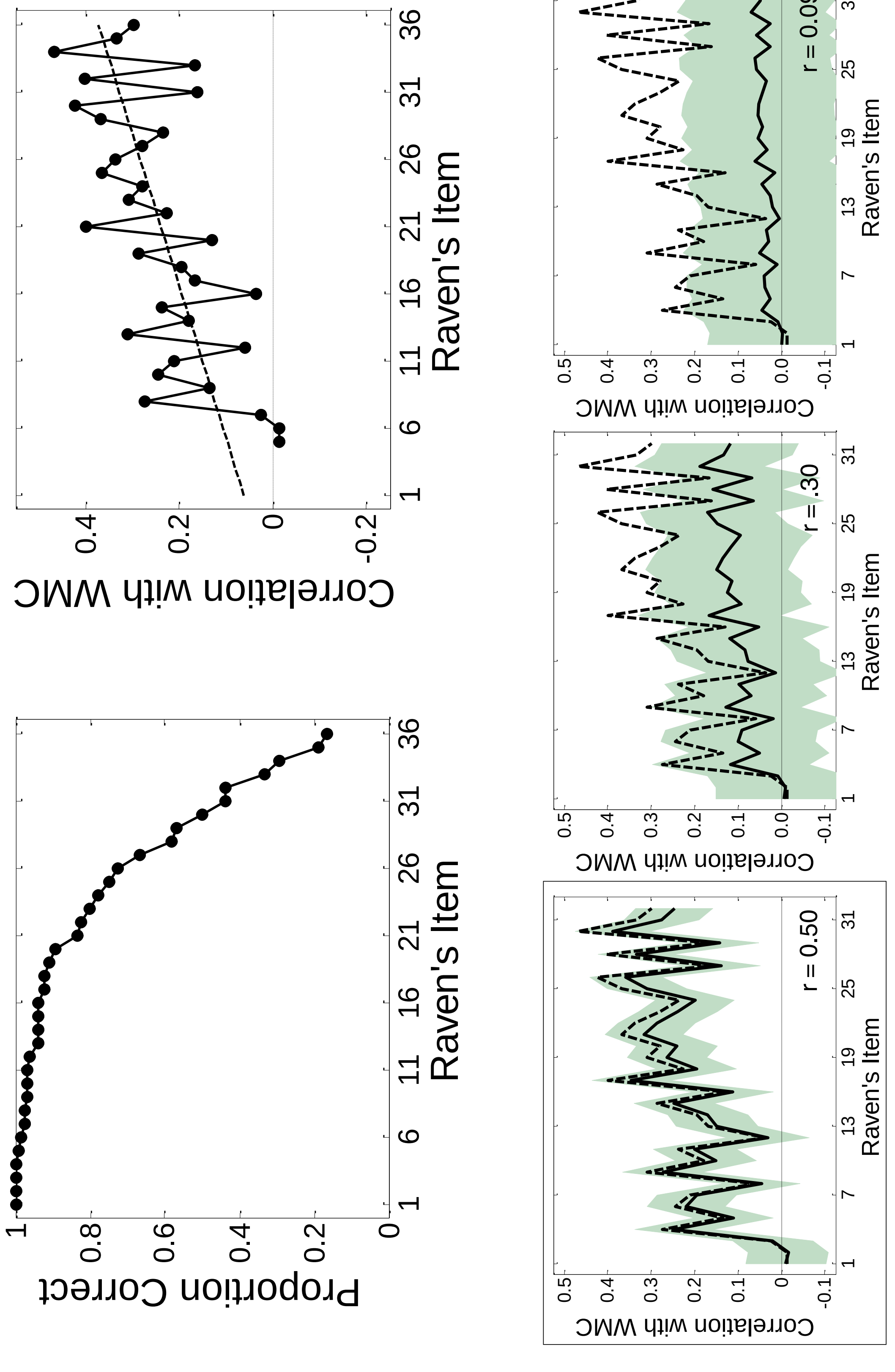

Proportion Correct
Raven's Item
Correlation with WMC
Raven's Item
Correlation with WMC
Raven's Item
r = 0.50
Correlation with WMC
Raven's Item
r = .30
Correlation with WMC
Raven's Item
r = 0.09
